# To B (Bone Morphogenic Protein-2) or Not to B (Bone Morphogenic Protein-2): Mesenchymal Stem Cells May Explain the Protein’s Role in Osteosarcomagenesis

**DOI:** 10.3389/fcell.2021.740783

**Published:** 2021-11-17

**Authors:** Chunfeng Xu, Mingjie Wang, Behrouz Zandieh-Doulabi, Wei Sun, Lingfei Wei, Yuelian Liu

**Affiliations:** ^1^Department of Oral Cell Biology, Academic Center for Dentistry Amsterdam (ACTA), University of Amsterdam and Vrije Universiteit Amsterdam, Amsterdam, Netherlands; ^2^Department of Mechanical Engineering, Drexel University, Philadelphia, PA, United States; ^3^Department of Mechanical Engineering, Tsinghua University, Beijing, China; ^4^Department of Oral Implantology, Yantai Stomatological Hospital, Yantai, China

**Keywords:** osteosarcoma, bone morphogenetic protein-2, mesenchymal stem cells, osteogenic differentiation, osteogenesis, tumor heterogeneity, bone-marrow-derived mesenchymal stem cell

## Abstract

Osteosarcoma (OS), a primary malignant bone tumor, stems from bone marrow-derived mesenchymal stem cells (BMSCs) and/or committed osteoblast precursors. Distant metastases, in particular pulmonary and skeletal metastases, are common in patients with OS. Moreover, extensive resection of the primary tumor and bone metastases usually leads to bone defects in these patients. Bone morphogenic protein-2 (BMP-2) has been widely applied in bone regeneration with the rationale that BMP-2 promotes osteoblastic differentiation of BMSCs. Thus, BMP-2 might be useful after OS resection to repair bone defects. However, the potential tumorigenicity of BMP-2 remains a concern that has impeded the administration of BMP-2 in patients with OS and in populations susceptible to OS with severe bone deficiency (e.g., in patients with genetic mutation diseases and aberrant activities of bone metabolism). In fact, some studies have drawn the opposite conclusion about the effect of BMP-2 on OS progression. Given the roles of BMSCs in the origination of OS and osteogenesis, we hypothesized that the responses of BMSCs to BMP-2 in the tumor milieu may be responsible for OS development. This review focuses on the relationship among BMSCs, BMP-2, and OS cells; a better understanding of this relationship may elucidate the accurate mechanisms of actions of BMP-2 in osteosarcomagenesis and thereby pave the way for clinically safer and broader administration of BMP-2 in the future. For example, a low dosage of and a slow-release delivery strategy for BMP-2 are potential topics for exploration to treat OS.

## Introduction

Although osteosarcoma (OS), a primary bone neoplasm, is rare, with an incidence of only one to three confirmed cases per 1 million people in the world each year, it comprises ∼20% of newly diagnosed bone tumors (Dorfman and Czerniak, 1995; Klein and Siegal, 2006; Mirabello et al., 2009b). Epidemiologically, OS presents in children, the youth, and the elderly with high frequency (Kansara et al., 2014); the morbidity of OS increases to 8–11 per million annually in 15–19-year-olds (Stiller et al., 2006; Mirabello et al., 2009a; Anfinsen et al., 2011). OS most often initiates in the metaphysis of long bones (Ritter and Bielack, 2010), implying a correlation with impaired bone growth. Currently, bone-marrow-derived mesenchymal stem cells (BMSCs) and/or committed osteoblast precursors with genomic mutations (e.g., *TP53*, *RB1*), chromosomal deletion, and chromosomal rearrangements are recognized as the cellular origins of OS (Chandar et al., 1992; Walkley et al., 2008; Mohseny et al., 2009; Rubio et al., 2013; Chen et al., 2014; Deng et al., 2019; Han et al., 2019). As an aggressive tumor, OS is insensitive to some chemotherapy agents (Pavlou et al., 2019; Belisario et al., 2020); MAP (methotrexate, doxorubicin, and cisplatin) is still the first-line drug for OS chemotherapy (Marina et al., 2016). To date, the OS has a 5-year survival rate of ∼50% (Smeland et al., 2019); the leading cause of death in OS is pulmonary metastasis (Bhattasali et al., 2015). Skeletal metastasis is also common in patients with OS and precipitates severe bone erosions. Extensive resection to remove OS is also responsible for voluminous bone defects, which may induce dysfunction and disfiguration. The rehabilitation of bone tissue is a huge challenge in clinical OS therapy. Although bone morphogenic protein-2 (BMP-2) has been widely used in bone repair and has shown promising results, its application in OS has not been reported because of its potential role in tumorigenesis.

BMP-2 was discovered by Urist (1965), and its cDNA was first cloned by Wozney et al. (1988). This growth factor is a member of bone morphogenic proteins (BMPs) belonging to the transforming growth factor-beta (TGF-β) superfamily that is important for diverse cellular processes (e.g., cell proliferation, differentiation, apoptosis, angiogenesis, migration, and extracellular matrix remodeling) (Bierie and Moses, 2006; Massagué, 2008). More than 20 BMPs have been identified in human tissues (Wozney and Rosen, 1998; Reddi, 2005). As the most well-studied one, BMP-2 has been widely used in bone formation because of its potent osteoinductivity and has been approved by the U.S. Food and Drug Administration for orthopedic and dental applications (Burkus et al., 2002; US Food and Drug Administration, 2002; Alonso et al., 2014). After continued clinical use, the adverse effects of BMP-2 (e.g., inflammatory, ectopic bone formation, infection, and potential tumorigenicity) have come into focus; the high dose and off-label application of BMP-2 have also aroused concern (Cahill et al., 2009; Tian et al., 2017; Pardali et al., 2018; Hashimoto et al., 2020; Hsu et al., 2020). Whether BMP-2 suppresses or stimulates tumor development remains a contentious issue (Weiss, 2015), and this controversy still challenges researchers (Kendal et al., 2020; Table 1). Using an orthotopic mouse model, Xiong et al. (2018) revealed that 2.5 μg of recombinant human BMP-2 (rhBMP-2) applied for 14 days not only induced bone formation but also suppressed OS growth and pulmonary metastasis in OS-bearing mice. Similar research also documented that rhBMP-2 constrained the tumorigenicity of cancer stem cells in human OS cell lines *in vitro* and *in vivo* (Wang et al., 2011; Gill et al., 2017). Conversely, opposite results from other studies have suggested that BMP-2 promotes OS migration and epithelial–mesenchymal transition (Sotobori et al., 2006; Tian et al., 2019). Because of this discrepancy in results, the use of BMP-2 in those at high risk of OS must be discreet and individualized based on the latest research.

**TABLE 1:** Studies of bone morphogenic protein 2 on tumor progression.

**References**	**Animal model**	**Cell lines**	
Xiong et al. (2018)	Mice	143B	Lung metastasis↓, Ki-67 ↓, ALDH^br^↓
Rampazzo et al. (2017)	NA	GBM- derived cells	Ki67↓, drug susceptibility↑, differentiation of GSCs↑
Wang et al. (2012)	Mice	ACHN, Caki-2	Tumor proliferation↓, Runx2↑, tumor volume↓, bone formation↑
Nishimori et al. (2012)	NA	LNCaP, MC3T3-E1	FGF-2↑, EGF↑ LNCaP cells proliferation↑
Kang et al. (2011)	NA	AGS, SNU-638	Cell migration and invasion↑, NF-κB activity↑, MMP-9↑
Wu J. B. et al. (2011)	NA	SMMC7721	Cell invasion↑, MMP-2 and MMP-9↑, p-ERK↑

*ALDH^br^, aldehyde dehydrogenase bright; EGF, epidermal growth factor; FGF-2, fibroblast growth factor (FGF)-2; GBM, glioblastoma; GSCs, glioblastoma stem cells; NA, not available.*

**FIGURE 1:**
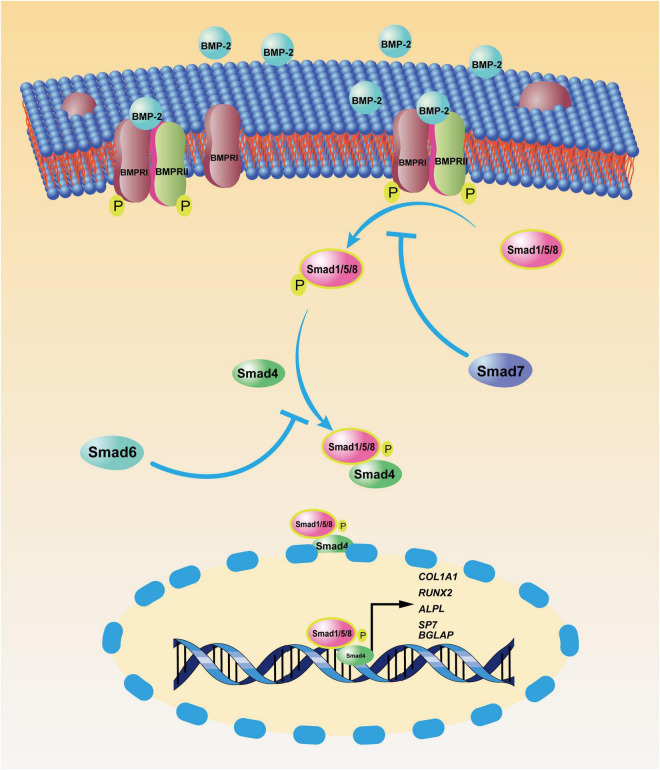
Canonical bone morphogenic protein-2 (BMP-2) signaling pathway. After BMP-2 binds to its transmembrane receptors [bone morphogenic protein receptor (BMPR)I and BMPRII], these phosphorylated receptors facilitate the phosphorylation of mothers against decapentaplegic and the *Caenorhabditis elegans* protein 1/5/8 (Smad1/5/8) in the cytoplasm. Then, the complex of pSmad1/5/8 and Smad 4 translocates to the nucleus, where phosphorylated Smad1/5/8 (pSmad1/5/8) and Smad 4 function as transcription factors, enhancing the transcription of osteoblastic genes, including *COL1A1, RUNX2, ALPL, SP7*, and *BGLAP*. As negative feedback, Smad7 inhibits the phosphorylation of Smad1/5/8, and Smad6 prevents the nucleus translocation of the complex of pSmad1/5/8 and Smad4.

Mesenchymal stem cells (MSCs) are identified as the origin of OS and are capable of differentiating into osteoblasts, a process that can be accelerated by BMP-2. However, BMP-2 is also involved in the progression of OS, suggesting that complicated crosstalk may exist among OS, BMP-2, and MSCs. As multipotent mesenchymal stromal cells, MSCs are universally found in almost all connective tissues (Horwitz et al., 2005; da Silva Meirelles et al., 2006). They possess the ability to differentiate into various mature somatic cells (e.g., osteoblasts, adipocytes, and chondrocytes) with appropriate stimulation (Pittenger et al., 1999) and the capacity to self-renew. BMSCs were first isolated by Owen and Friedenstein (1988) from bone marrow. These heterogeneous cells are involved in osteoblast differentiation through the spatiotemporal expression of osteogenesis-related genes (*RUNX2, COL1A1, ALPL, SP7, BGLAP*, etc.) (Ducy et al., 1997; Nakashima et al., 2002; Twine et al., 2014). A few signal pathways have proven to have pivotal roles in BMSC-induced osteogenesis; the canonical BMP-2 pathway (Figure 1) is a well-known example. A great body of research has focused on the effect of MSCs on or toward osteoblastic differentiation and OS progression; to date, though, the effect of BMP-2 on normal BMSCs and on mutated BMSC–induced osteosarcomagenesis is still elusive.

MSCs play contradictory roles in copious cancer types (Devarasetty et al., 2017; Gyukity-Sebestyén et al., 2019; Xu et al., 2019; Zhang et al., 2020; Jia et al., 2021). In OS, MSCs are reportedly involved in not only chemoresistance, proliferation, and pulmonary metastases but also OS recession (Cortini et al., 2017). Thus, the effect of MSCs on OS might be converted according to the relevant OS niche. Herein, we summarize the literature and present the potential mechanism of the contradictory effects of MSCs on OS to provide direction for additional studies.

## Bone Morphogenic Protein-2 Inhibits Osteosarcoma Progression *via* Mesenchymal Stem Cells

**FIGURE 2:**
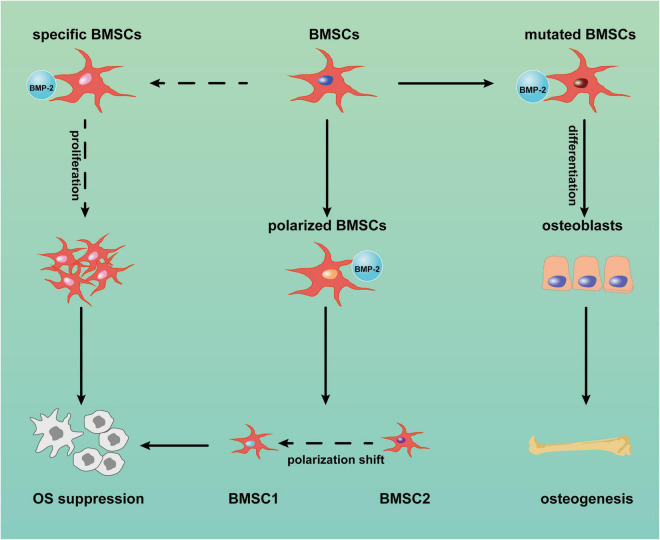
Potential mechanism of bone morphogenic protein-2 (BMP-2) induced tumor suppression *via* bone marrow-derived mesenchymal stem cells (BMSCs). BMP-2 may induce mutated BMSC differentiation into normal osteoblasts. Conversely, BMP-2 may promote the proliferation of specific BMSCs with anticancer capacity and the shift of BMSC polarization from MSC2 (tumor promotion) to MSC1 (tumor inhibition). OS: osteosarcoma.

MSCs can suppress sarcoma progression. Gauthaman et al. (2012) found that umbilical cord-derived MSCs from Wharton’s jelly suppressed the proliferation and migration of MG-63 cells (a human OS cell line) *in vitro*; in a Kaposi sarcoma model, MSCs also inhibited tumor progression (Khakoo et al., 2006). BMP-2 also has inhibited OS progression, although the potential mechanism was not discussed (Xiong et al., 2018). Given the close link between MSCs and BMP-2 in osteoblastic differentiation and OS etiology, BMP-2 might suppress OS through BMSCs. We reviewed the literature to explore the ability of BMP-2 to inhibit OS through BMSCs and present three assumptions (Figure 2): (1) BMP-2 induces proliferation of BMSCs with the capacity to suppress OS; (2) BMP-2 induces differentiation of mutated BMSCs and/or OS cells to normal osteoblasts; (3) BMSC polarization shifts.

### Proliferation of Specific Mesenchymal Stem Cells

**FIGURE 3:**
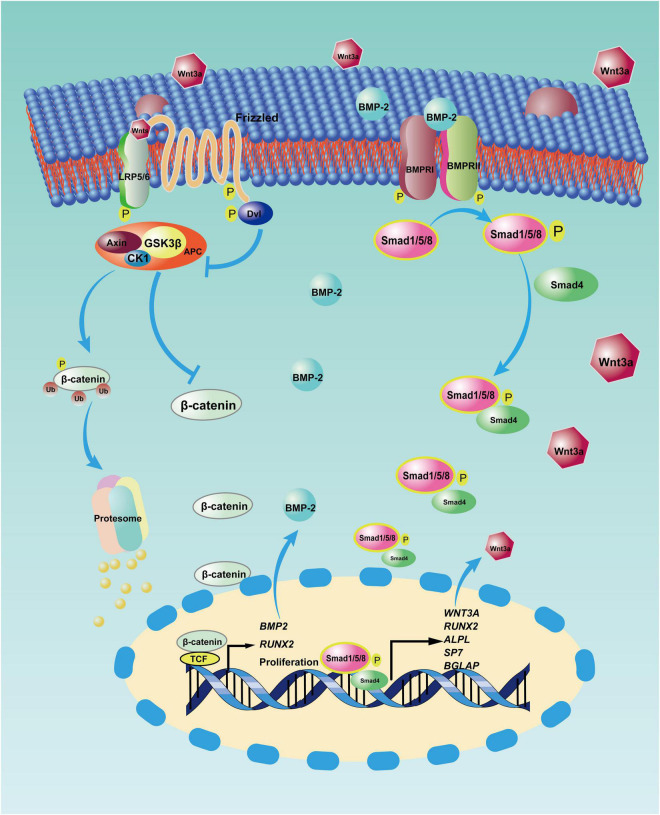
Reciprocal activation between canonical bone morphogenic protein-2 (BMP-2) and canonical Wnt signaling pathway. After BMP-2 binds to its receptors on the cell surface, phosphorylated Smad1/5/8 and Smad4 translocate into nuclei, where this complex modulates the transcription of some target genes, including *WNT3A* (coding the Wnt3a protein) and some osteoblastic differentiation genes. However, with the autocrine signaling, Wnt3a binds to its receptors (i.e., LRP5/6 and Frizzled) on the membrane, so β-catenin accumulates in the nuclei and incorporates with T cell-specific transcription factor (TCF) to upregulate the transcription of target genes, including *BMP2, RUNX-2*, and proliferation-related genes. APC: adenomatous polyposis coli, CK1: casein kinase 1, Dvl: Disheveled, GSK3β: glycogen synthase kinase3β, LRP5/6: low-density lipoprotein receptor-related protein 5.

BMSCs are heterogeneous populations comprising various subpopulations with diverse properties (Horwitz et al., 2005). Except for Wharton’s jelly MSCs, BMSCs from rats and mice have demonstrated dose-dependent cytotoxicity to tumor cells (Otsu et al., 2009). Thus, specific BMSCs with anticancer capacity exist and may function according to the altered expression of some cytomembrane receptors (Ridge et al., 2017). It would make sense that BMP-2 could suppress OS through the proliferation of these specific BMSCs and that a BMP-2 and Wnt pathway autocrine loop (Figure 3) may be capable of explaining this process. The Wnt pathway is involved in diverse cellular events, including mitogenic stimulation, cell fate determination, differentiation, and proliferation (Huang and Niehrs, 2014; Yao et al., 2016; Steinhart and Angers, 2018). It is not surprising that the Wnt pathway, in particular the canonical Wnt pathway (i.e., the beta-catenin–dependent pathway), plays crucial roles in osteoblastic differentiation and osteogenesis (Liu et al., 2008; Wang et al., 2014b; Lerner and Ohlsson, 2015). Although the Wnt pathway is thought to inhibit MSC proliferation (Moon et al., 2018), an activated Wnt pathway facilitating BMSC proliferation has also been reported (Zhu et al., 2014). After the canonical Wnt pathway is activated, beta-catenin translocates from the cytoplasm into nuclei. In the nuclei, a complex consisting of beta-catenin and some transcription factors—for example, lymphoid enhancer-binding factor 1/T cell-specific transcription factor (LEF/TCF)—modulates the expression of target genes, including *BMP2*, *RUNX2*, and proliferation-related genes (Zhang R. et al., 2013). Conversely, BMP-2 can stimulate the accumulation of beta-catenin in nuclei (Yang et al., 2006; Hiyama et al., 2011), thereby activating the canonical Wnt pathway in turn. BMP-2–induced cell proliferation has been reported in murine preosteoblasts, rat BMSCs, and human pulmonary artery epithelial cells (de Jesus Perez et al., 2009; Rosen, 2009; An et al., 2017). The OS suppression properties of BMP-2 might result from the positive feedback of this loop *via* expansion of the specific BMSCs in the OS niche. In addition, aberrant activation of Wnt/beta-catenin signaling in OS cells has been detected (Chen et al., 2015). The identification of specific BMSCs in the OS niche is a precondition for OS suppression. Unfortunately, few studies about these specific BMSCs have been conducted in OS settings, so detailed information about their characteristics is still lacking.

### Induced Osteoblastic Differentiation

Cancer is a disease arising from failed cell differentiation (Honma and Akimoto, 2007). Thus, differentiation-inducing treatments have been proposed. With this strategy, tumor cells differentiate back into normal cells instead of being eliminated by chemotherapeutics and/or radiation. One well-known differentiation-inducing treatment is all-trans-retinoic acid in acute promyelocytic leukemia (Huang et al., 1988). Notably, OS is recognized as an osteoblast differentiation disruption disease (Tang et al., 2008). OS cells have characteristic properties that resemble undifferentiated osteoblasts (Carpio et al., 2001; Postiglione et al., 2003; Haydon et al., 2007), and activating *RB1* transcription has reversed the disrupted osteoblastic differentiation (Thomas et al., 2001). In addition, BMP-2 has been tested for its efficacy as a differentiation-inducing treatment. Rampazzo et al. (2017) successfully induced astroglial differentiation of glioblastoma stem cells using a BMP-2 mimicking peptide. Moreover, BMP-2 has suppressed tumors and promoted bone formation simultaneously: Wang et al. (2012) indicated that renal cell cancer was inhibited and bone formation was induced with the application of BMP-2. Furthermore, BMP-2 has reduced tumor volume, attenuated OS-induced pulmonary metastases, and stimulated bone formation (Xiong et al., 2018). Applying 30 μg of BMP-2 to OS-bearing mice also increased the transcription of osteogenic genes and promoted osteogenesis (Wang et al., 2013). Taken together, these data suggest that BMP-2 may play a therapeutic role in OS by inducing osteogenic differentiation of mutated BMSCs and/or OS cells.

### Mesenchymal Stem Cell Polarization

The polarization of macrophages in inflammatory conditions suggests that the effect of BMSCs on OS may also transform mutually between tumor promotion and tumor suppression (Ridge et al., 2017). This hypothesis has been verified by Waterman et al. (2012) in a study that activated different cytomembrane receptors. The researchers claimed that activation of toll-like receptor-4 (TLR-4) conferred an antitumor effect on human BMSCs, which were named MSC1; after TLR-3 activation, however, the human BMSCs were converted to MSC2, which promoted tumor growth and metastasis (Waterman et al., 2012). Although myriad studies have indicated that TLR-2 and TLR-4 can enhance the expression of BMP-2 in BMSCs and accelerate bone formation (Yang et al., 2009; Su et al., 2011; Oliveira et al., 2017; Zhou et al., 2019), the effect of BMP-2 on the expression of TLRs is still equivocal. The dosage of BMP-2 and the state of BMSCs in the tumor niche may draw contrasting conclusions. Thus, the hypothesis that BMP-2 suppresses OS by affecting TLRs must be explored in more detail.

## Bone Morphogenic Protein-2 Promotes Osteosarcoma Progression *via* Mesenchymal Stem Cells

### Aberrant Activation of *RUNX2* and *SP7*

**FIGURE 4:**
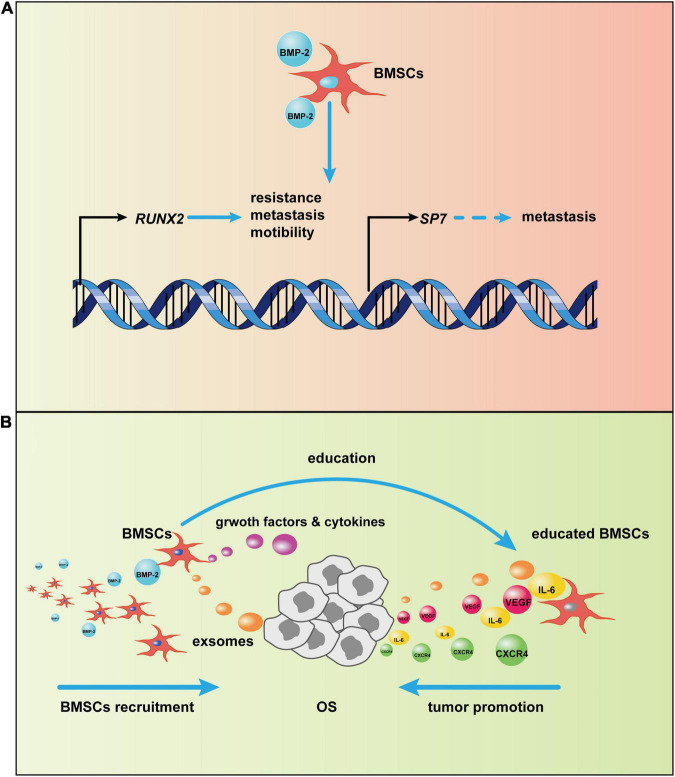
Potential mechanism of bone morphogenic protein-2 (BMP-2)–induced tumor progression *via* bone marrow-derived mesenchymal stem cells (BMSCs). **(A)** After the canonical BMP-2 pathway is activated, *RUNX2* and *SP7* transcription initiate. The overexpression of *RUNX2* and *SP7*, as a result of continuous activation of the canonical BMP-2 pathway, may promote osteosarcoma (OS) progression. **(B)** BMSCs are recruited to OS by BMP-2. Then, BMSCs adapt to OS *via* OS-related cytokines and exosomes; in turn, the tumor-centered BMSCs will secrete growth factors and cytokines, such as vascular endothelial growth factor (VEGF) and CXC chemokine receptor 4 (CXCR4), to promote OS development.

More research has reported that BMPs, especially a supra-physiological dose of BMP-2, induces tumorigenesis, not tumor suppression (Figure 4; Jin et al., 2001; Kang et al., 2011; Wu J. B. et al., 2011; Nishimori et al., 2012; Tian et al., 2017; Zhang et al., 2018). The physiological concentration of BMP is ∼2 ng/g of bone. In most clinical trials, supra-physiological doses (mg concentrations) of BMP-2 have been applied, and these doses may disturb the normal BMP-2 signal pathway (Arrabal et al., 2013; Oryan et al., 2014). After BMP-2 binds to its receptors on the cell surface, the BMP-2 signaling pathway is activated. In the canonical BMP-2 pathway, transcription of osteogenic genes, including *RUNX2*, and *SP7* (*OSTERIX*), is upregulated. Although these two genes are vital for bone formation, an increasing body of evidence implies that they are also engaged in tumorigenesis. Normally, *RUNX2* expresses during the cell cycle in healthy osteoblasts to disturb cell growth and induce osteoblast maturation (Pratap et al., 2003). Overexpression of *RUNX2* has been found in patients with OS and is correlated to poor prognosis (Pereira et al., 2009; Sadikovic et al., 2010; Gupta et al., 2019). van der Deen et al. (2012) used chromatin immunoprecipitations to detect *RUNX2* target genes in U2OS cells; results indicated that some motility-related genes were downstream of *RUNX2* and that cell motility decreased after *RUNX2* depletion. Furthermore, an elevated RUNX2 protein level may also be responsible for pulmonary metastasis. After *RUNX2* activates *SPP1* (*OPN*), the *RUNX2* target gene encodes a secreted matricellular protein, thereby remodeling the bone matrix, which leads to tumor metastasis (Villanueva et al., 2019). *RUNX2* may also account for the chemotherapeutic resistance of OS. When *RUNX2* was silenced by si/shRNA, OS cells were more sensitive to doxorubicin (Roos et al., 2015). Another osteogenic gene, *SP7*, has not been associated with osteosarcomagenesis, but it has been described as a stimulus in other tumors (Dai et al., 2015; Yao et al., 2019; Ricci et al., 2020). This finding suggests that sustained activation of the BMP-2 pathway causing increased *SP7* transcription may also precipitate OS in bone tissues.

### Modulation of the Tumor Microenvironment

BMP-2 may promote OS progression through modulation of the tumor microenvironment (TME), which plays an indispensable role in tumor progression (Hui and Chen, 2015; Yang et al., 2020). The bone microenvironment where OS grows is composed of hematopoietic stem cells, lymphoid progenitors, mature immune cells, bone cells, MSCs, mineralized extracellular matrix, and more (Tsukasaki and Takayanagi, 2019; Corre et al., 2020). The crosstalk in these items modulates the OS TME, which affects OS progression. Cancers are identified as “wounds that never heal” (Dvorak, 1986), so it is not surprising that MSCs are involved in tumor development, given the central role of MSCs in repairing wounds by altering the local inflammatory environment and secreting growth factors, immunoregulatory factors, and chemokines (Caplan and Correa, 2011; Wang et al., 2014a; Shi et al., 2017) after the tumor-specific tropism of MSCs (Kidd et al., 2009). However, MSCs are not always beneficial for healing; the fluctuation of their function depends on the milieu where they reside (Wang et al., 2014a). In the TME, MSCs can be converted into tumor-associated MSCs that have vast differences from normal MSCs (Le Nail et al., 2018) and that can promote tumor proliferation, migration, immunosuppression, and angiogenesis through extracellular vesicles (Quante et al., 2011; Baglio et al., 2017; Shi et al., 2017; Whiteside, 2018). In the OS niche, interleukin-6 (IL-6) and vascular endothelial growth factor (VEGF) secreted from BMSCs have been involved in OS progression (Tu et al., 2012; Zhang P. et al., 2013); BMSCs promoted pulmonary metastasis of OS by increasing the expression of CXC chemokine receptor 4 (CXCR4) and VEGF (Fontanella et al., 2016). Furthermore, extracellular vesicles, such as exosomes from BMSCs, are loaded with certain miRNAs involved in OS aggression and development (Xie et al., 2018). BMP-2, as a member of the TGF-β superfamily with the ability to recruit MSCs to inflammatory surroundings and the TME (Spaeth et al., 2008), may recruit BMSCs to OS, and BMP-2-induced chemotaxis has been reported in other conditions (Hiepen et al., 2014; Simões Sato et al., 2014; Pardali et al., 2018). BMP-2, particularly at high doses, induces inflammation (James et al., 2016), which may cause MSC homing as a result of inflammatory cytokines; in addition, MSCs have been recruited by BMP-2 through CXCR4, accelerating bone formation (Zwingenberger et al., 2014). Thus, BMP-2 might recruit BMSCs toward the OS phenotype. Together, these results suggest a tentative hypothesis. After BMSCs are recruited by BMP-2 to the OS niche, they will be educated directly or indirectly by OS cells. Afterward, the emergence of the educated BMSCs that can secrete some cytokines and growth factors will promote OS proliferation, migration, angiogenesis, and more.

## Reasons for Contradictory Conclusion

The debate about BMP-2 is an obstacle to its clinical application, despite the potential value for those at high risk of OS and for patients with OS and bone defects. Illustrating the reasons for these controversies can deepen our understanding of the function of BMP-2 in OS and guide its clinical administration.

### Differences in Osteosarcoma Cell Lines

Diverse OS cell lines applied in the research contribute to the confusion about results. Histologically, several OS subtypes with distinct characteristics have been confirmed. At the cellular level, various *in vitro* OS cell lines have been used in research; great differences in these cell lines have been verified. Saos2 cells appear more identical to normal osteoblasts than other OS cell lines, as osteoblastic markers can be detected in these cells. Conversely, osteocalcin, an important marker in bone mature, was hardly expressed in MG-63 and U2OS cells. However, matrix metalloproteinase-9 (MMP-9), a well-known cytokine for tumor migration and metastasis (Deryugina and Quigley, 2006), was positive in most MG-63 cells (Pautke et al., 2004). In other research, researchers (Mohseny et al., 2011) compared differences in differentiation, tumorigenesis, and protein expressions among 19 OS cell lines. Only OSA, IOR/OS9, and IOR/OS18 could differentiate into osteoblasts, chondrocytes, and adipocytes; 13 of the 19 cell lines could differentiate toward osteoblasts. This finding may explain why some researchers claimed that OS cell lines could not be induced into osteoblasts by BMP-2, whereas other studies reported opposite results (Haydon et al., 2007). Moreover, in these 19 OS cell lines, HOS-14B cells had the greatest capacities of tumorigenesis and metastasis. These inherent disparities between various OS cell lines, to some extent, account for the conflicting conclusions about the role of BMP-2 in OS progression.

### Heterogeneity of Mesenchymal Stem Cells

Variations in MSCs are also ubiquitous. MSCs are heterogeneous populations consisting of a few subtypes with diverse characteristics; the differences may come from individual differences and species differences (Peister et al., 2004). The proposed definition of MSCs suggests that they must (1) adhere to plastic, (2) express special surface markers, and (3) differentiate along the osteogenic, chondrogenic, and adipogenic lineages (Dominici et al., 2006; Lindsay and Barnett, 2017). Commonly, CD34, CD31, and CD45 are negative on both human and mouse MSCs (Dominici et al., 2006); some markers, such as STRO-1 and CD271, are only detected on human MSCs (Lv et al., 2014); these are specific and can be found on other cell types (Kuhn and Tuan, 2010). CD29, CD51, CD73, CD90, CD105, and CD146 are universal in human and mouse MSCs (Sacchetti et al., 2007; Zhang et al., 2019). BMSCs are the most used MSCs in research; they are heterogeneous as well, which complicates the research and weakens the conclusions. Although some specific isolation kits based on the cell surface markers have been applied to clarify results, it remains hard to purify the homogeneous BMSCs, as MSCs share cell-surface markers and localization with pericytes (Crisan et al., 2008). With the development of biotechnology, the function and characteristic identification of a single cell are practicable. Single-cell RNA sequencing has been used to detect immune cell heterogeneity (Papalexi and Satija, 2018); Zhou et al. (2020) assayed the intratumoral heterogeneity and immunosuppressive microenvironment in advanced OS and demonstrated the complex variations in OS.

### Different Doses and Delivery Strategies of Bone Morphogenic Protein-2

Furthermore, the dose and the delivery strategy of BMP-2 affect the research conclusions (Wu G. et al., 2011). Most of the reported disadvantages of BMP-2 result from overdosage. The effective dose of BMP-2 in osteoblastic differentiation of MSCs, which is dose-dependent, is just 25–100 ng/mL *in vitro* (Rickard et al., 1994; Lecanda et al., 1997). However, the working concentration of BMP-2 for *in vitro* or *in vivo* research is not distinguished, and most doses are supra-physiological, which may confound the results and cause adverse effects. The delivery pattern of BMP-2 is also crucial. A continuous and slow release, rather than a burst stimulation, is more bionic and more closely resembles physiological conditions. Most recent research has administered rhBMP-2 protein directly into the culture medium or intravenously, which may cause stress conditions for cells and tissues. The advantages of a sustained, low-dose release of BMP-2, including less inflammation and ectopic ossification, have been verified (Wildemann et al., 2004; Ji et al., 2010; Seo et al., 2017; Berkmann et al., 2020; Xin et al., 2020). The mitigatory inflammatory surroundings can reduce the risk of tumorigenesis as well, which makes low-dose BMP-2 application more reasonable.

## Limitations in Present Studies

### Deficiency of *in vitro* Research

Currently, most *in vitro* studies are carried out on traditional two-dimensional (2D) culture models (i.e., flask- and petri-dish-based cultures). However, these 2D models hardly mimic tumor cell biology because of tumor heterogeneity and different responses to secreted cytokines, growth factors, and methylation states of the cells. Moreover, the 2D cell culture systems cannot sufficiently simulate a three-dimensional (3D) physiological microenvironment, so they fail to provide physiologically relevant information regarding cell–cell interactions, cell–extracellular matrix interactions, growth factor synthesis, or physical and chemical cues to oncogenesis (Hickman et al., 2014; Berkmann et al., 2020). Furthermore, the results obtained from gene expression analysis and drug resistance also differ substantially between 2D and 3D cell culture models (Zhao et al., 2014; Costa et al., 2016; Henriksson et al., 2017; Zhou et al., 2017; Fontoura et al., 2020; Mao et al., 2020; Sun et al., 2020; Xie et al., 2021). The disadvantages of the 2D culture reduce attempts to understand the authentic role of BMP-2 that may play in the formation and pathology of OS.

### Inappropriate Animal Models

In most OS studies, rodents, such as mice or rats, have been used as experimental animal models in addition to the patient-derived xenograft or cell line-derived xenograft models. Normally, OS is rare in mice and rats, and these models may present limited information or misinformation. The OS incidence in dogs is ∼27-fold higher than in humans, which makes the canine model a more useful model to the human OS for research (Simpson et al., 2017). To date, preclinical research using dogs as animal models has suggested that a combination of canine BMSCs together with rhBMP-2 treatment suppressed OS by increasing p53 and some other pro-apoptotic proteins (Rici et al., 2012, 2018). However, using dogs as animal models to study the effects of BMP-2 on OS development is not well accepted in Western countries because of social and cultural reasons.

### Lack of High-Quality Evidence

Large-scale and multicenter cohort studies for evaluating BMP-2 treatment effects on OS progression remain unavailable. Although some clinical retrospective studies have suggested that BMP-2 used in spine fusion surgery was not involved in tumorigenesis (Fahim et al., 2010; Cooper and Kou, 2013; Lad et al., 2013), these studies were performed with small sample sizes and had insufficient follow-up times. Large-scale and multicenter cohort studies are needed to draw a scientific conclusion and establish the effects of the BMP-2 on patients living with cancer.

## Summary

To date, the exact role of BMP-2 in osteosarcomagenesis is still equivocal, although abundant studies have been carried out. This uncertainty is attributed to the intricacy of the OS genome, differences between OS subtypes, the complex TME, and the multifunctionality of BMP-2 activation of several signal transduction pathways. The response of MSCs, which have a pivotal effect on osteogenesis and osteosarcomagenesis, to BMP-2 remains a key to understanding this mystery. This review represents research focused on the BMP-2 effect on OS cell lines and OS animal models and the relevant potential mechanisms involved, and it provides some clues for additional research about OS biology and safe application of BMP-2 in clinical settings. For current clinical application, we recognize that a low-dose and slow-release strategy of BMP-2 applied in bone regeneration is acceptable, even in the tumor-caused bone defects, while in the OS treatment, we still maintain a prudent stand to the employment of BMP-2.

As a growth factor, BMP-2 plays a crucial role in various cell biology activities. BMP-2 use in populations with genetic mutation diseases may promote OS progression; mutations of some genes, particularly *TP53* and *RB1*, and genomic alterations have been associated with osteosarcomagenesis. Likewise, using BMP-2 in patients with some bone metabolic diseases might increase the occurrence of OS, because aberrant activities of osteogenesis-related signaling pathways in these patients are very common; these pathologic activities may enhance the expression of *RUNX2* and *SP7*, the latter of which is overexpressed in patients with OS and is correlated with poor prognosis.

However, BMP-2 is highly likely to be used in OS treatments because of the BMP-2-induced proliferation of specific BMSCs with anticancer capacity. This strategy is based on the isolation and identification of these specific BMSCs. However, to our knowledge, no research on the isolation and identification of characteristics of these specific BMSCs has been carried out. Moreover, BMP-2 may inhibit OS through the osteoblastic differentiation of OS cells and/or mutated BMSCs. In addition, in line with the current consensus, although an overdose of BMP-2 could lead to over-proliferation of cells, which may increase the risk of neoplasm formation and tumorigenesis, using a low dose and a slow-release delivery pattern of BMP-2 appears safe for oncogenesis-related research.

For additional investigations, researchers should pay attention to the differences between various OS cell lines and the diverse OS subtypes. These differences are responsible for the contradictory roles of BMP-2 in OS development. Caution is needed to interpret data about the function of BMP-2 in OS progression when only one subtype of OS cell line is investigated. Because of the various limitations and factors involved, the relationship between BMP-2—in particular, the supra-physiological concentration of BMP-2—and OS has not been determined thoroughly; more research in this field is necessary.

## Author Contributions

CX contributed to the conception of this work and drafted the manuscript. MW performed literature search. BZ-D made important revisions. WS revised this manuscript and polished language. LW revised this manuscript and supplemented it with important information. YL edited and revised this manuscript and ultimately approved the publication. All authors contributed to the article and approved the submitted version.

## Conflict of Interest

The authors declare that the research was conducted in the absence of any commercial or financial relationships that could be construed as a potential conflict of interest.

## Publisher’s Note

All claims expressed in this article are solely those of the authors and do not necessarily represent those of their affiliated organizations, or those of the publisher, the editors and the reviewers. Any product that may be evaluated in this article, or claim that may be made by its manufacturer, is not guaranteed or endorsed by the publisher.
